# Trophic state (TSI_SD_) and mixing type significantly influence pelagic zooplankton biodiversity in temperate lakes (NW Poland)

**DOI:** 10.7717/peerj.5731

**Published:** 2018-10-05

**Authors:** Łukasz Sługocki, Robert Czerniawski

**Affiliations:** 1 Faculty of Biology, University of Szczecin, Szczecin, Poland; 2 University of Szczecin, Center of Molecular Biology and Biotechnology, Szczecin, Poland

**Keywords:** Species richness, Shannon index, Zooplankton, Rotifera, Cladocera, Copepoda, Mixing type, Lakes, Trophic state, Crustacea

## Abstract

**Background:**

Lake depth and the consequent mixing regime and thermal structure have profound effects on ecosystem functioning, because depth strongly affects the availability of nutrients, light, and oxygen. All these conditions influence patterns of zooplankton diversity. Zooplankton are a key component of the aquatic environment and are essential to maintaining natural processes in freshwater ecosystems. However, zooplankton biodiversity can be different regard to depth, mixing type and trophic state. Therefore, the aim of this study was to examine how depth and mixing regime affect zooplankton diversity in lakes. We also investigated the vertical distribution of diversity across a trophic gradient of lakes.

**Methods:**

A total of 329 zooplankton samples from 79 temperate lakes (36 polymictic and 43 dimictic) were collected. The biodiversity of zooplankton was calculated using species richness (SR) and the Shannon index (SI). An index based on Secchi disc visibility was used to determine the trophic state index (TSI_SD_) of lakes. The one-way ANOVA with Duncan’s post hoc test were used to determine differences in zooplankton biodiversity between mictic lake types and thermal layers. To find the best predictors for zooplankton biodiversity a multiple stepwise regression was used. The rarefaction method was used to evaluate the impact of mixing types, thermal layers, and the TSI_SD_on zooplankton biodiversity indices. A Sørensen similarity analysis and nonmetric multidimensional scaling (NMDS) were performed to describe the similarity patterns in species composition among lakes.

**Results:**

We identified a total of 151 taxa from 36 polymictic and 43 dimictic lakes. Lake depth and the TSI_SD_ were significantly correlated with the biodiversity of lake zooplankton. The results of ANOVA and Duncan tests show that mictic type and thermal zones had a significant effect on zooplankton biodiversity. The rarefaction curve showed significant differences in zooplankton biodiversity, which was greater in lakes with lower trophic state. Ordination by NMDS showed clustering of different mictic types, thermal layers, and composition changes throughout the TSI_SD_profile. Moreover, we determined that polymictic lakes are more heterogeneous than dimictic lakes in regard to zooplankton similarities.

**Discussion:**

Both mictic lake types were characterized by varying levels of zooplankton biodiversity, which is shaped by the communities’ response to lake depth, thermal layers and TSI_SD_ values. The zooplankton SR and SI (during daylight hours) depends greatly on the mixing type. Lake type also indicates the importance of the metalimnion in shaping zooplankton biodiversity in dimictic lakes. In addition, data from NW Polish lakes indicated that the increase of the TSI_SD_ leads to taxonomic shifts and has a negative effect on the diversity of all groups of zooplankton.

## Introduction

The mixing regime and thermal structure of lakes have a profound effect on ecosystem functioning because they strongly affect the availability of nutrients, light, and oxygen (Hutchinson, 1966; Wilhelm & Adrian, 2008; Gauthier, Prairie & Beisner, 2014). Polymictic lakes are shallow and mix to the bottom intermittently during the thawing period (Hutchinson, 1966; Shatwell, Adrian & Kirillin, 2016). In shallow lakes, regular mixing guarantees a stable light-dark cycle as well as higher heterotrophic activity, which stimulates primary production (Nixdorf & Deneke, 1997; Eleveld, 2012). Dimictic lakes are typically deep, mix only in spring and autumn, and stratify continuously over the warmer months (Kirillin & Shatwell, 2016). As was shown using a modeling approach, in dimictic lakes, a deep thermocline could decrease photoautotrophic production because the phytoplankton spends more time in darker waters, thus altering the productivity of aquatic organisms (Berger, Diehl & Kunz, 2006). In contrast, a greater thermocline depth could potentially boost the release of nutrients stored in the sediment, and in turn increase primary production (Scheffer & Van Nes, 2007). The diversity of mixing type regimes has implications for the biodiversity and composition of phytoplankton, macrophytes, macrobenthos, and ichthyofauna (Timms, 1982; Nixdorf, Mischke & Rücker, 2003; Grzybowski, 2014; Napiórkowska-Krzebietke & Hutorowicz, 2013; Czerniawski et al., 2015). Therefore, summer thermal stratification is also believed to be one of the key physical factors structuring the zooplankton communities of also for northern temperate lakes which was demonstrated in a whole lake experiment (Gauthier, Prairie & Beisner, 2014). Moreover, some authors suggest that changes in plankton communities may lead to mixing regime shifts in temperate lakes, which in turn can reveal the complexity between mixing regimes and aquatic organisms (Hambright, 1994; Shatwell, Adrian & Kirillin, 2016).

In both shallow and deep lakes, there are various mechanisms that can affect zooplankton biodiversity. Shallow lakes may be transparent and covered with macrophytes or turbid and dominated by phytoplankton (Scheffer & Van Nes, 2007). Shallow polymictic lakes usually present more diversity because of the array of microhabitats and refuge against predators (Manatunge, Asaeda & Priyadarshana, 2000; Meerhoff et al., 2007), as well as resources (Rautio & Vincent, 2006) offered by macrophytes. Phytoplankton is not the only source of food for the zooplankton. The proximity of the lake bottom (benthic resources) and mixing events increase the availability of dissolved organic matter, allochthonous materials, and bacteria for microinvertebrates (Rautio & Vincent, 2006; Mariash et al., 2014). Thus, there is expected to be a great diversity of Rotifera and Crustacea in polymictic lakes (Kuczyńska-Kippen, 2007; Magalhães Braghin, Simões & Bonecker, 2016). However, it would seem that polymictic lakes are unsuitable for certain zooplankton taxa if the lake conditions are not suitable for individual species (e.g., stenotherm species such as *Cyclops abyssorum*, *Daphnia hyalina, Eurytemora lacustris, Filinia terminalis, Heterocope appendiculata, Notholca squamula* or pH-sensitive species such as *Ascomorpha ovalis* (Radwan, 2004; Błędzki & Rybak, 2016). In dimictic lakes, a deep thermocline could decrease photosynthetic activities, altering the productivity of various zooplankton taxa or of the entire community (Berger, Diehl & Kunz, 2006; Gauthier, Prairie & Beisner, 2014). Less resource availability for various zooplankton taxa could lead to less diversity in the epilimnion, nonetheless many taxa could inhabit the deeper layers of lakes. However, in dimictic lakes with distorted stratification, the loss of a hypolimnetic refuge for large crustaceans is expected to occur (Maier et al., 2011; Gauthier, Prairie & Beisner, 2014).

Water transparency also has an impact on lake processes and the behavior of freshwater organisms (Fee et al., 1996; Berger, Diehl & Kunz, 2006). Transparency is considered a trophic state proxy (TSI_SD_) in water management and especially for ecological assessments (Carlson, 1977; Egan et al., 2009; Pyhälä, Fleming-Lehtinen & Laamanen, 2014; Stock, 2015; Binding et al., 2015; Heddam, 2016; Alikas & Kratzer, 2017). However, transparency is more than just an indicator. Transparent lakes are also an important supplier of viable water. Thus, transparency could affect zooplankton biodiversity. The variables that could cause a shift in zooplankton diversity (e.g., decreasing richness of sensitive species) in the deep layers of dimictic lakes, are anoxic conditions below the thermocline (Maier et al., 2011) or the presence of a large community of planktivorous fish (Gliwicz, 1986). These factors escalate the eutrophication process and decrease water transparency (Jeppesen et al., 2011). Rapid eutrophication often negatively affects the ecological balance of aquatic ecosystems and may lead to a decline in biodiversity (Dunne, Williams & Martinez, 2002; Dudgeon et al., 2006; Cardinale et al., 2012; Scherer & Pfister, 2016). Rotifer and crustacean communities are also affected by eutrophication, and therefore serve as indicators of changing ecological status (Karabin, 1985; Jeppesen et al., 2011; Ejsmont-Karabin, 2012; Ejsmont-Karabin & Karabin, 2013; Ochocka & Pasztaleniec, 2016). According to Ejsmont-Karabin (2012), relationships between zooplankton and trophic state are different between polymictic and dimictic lakes, and in both lake types rotifers are more responsive to trophic changes than crustaceans (Karabin, 1985; Ejsmont-Karabin & Karabin, 2013). When examining lake zooplankton biodiversity, authors often find a unimodal peak in species richness at intermediate primary productivity levels (Dodson, Arnott & Cottingham, 2000; Waide et al., 1999; Barnett & Beisner, 2007). However, Jeppesen et al. (2000) showed that zooplankton species richness declines along the eutrophication gradient of Danish lakes. That is, due to lake eutrophication, it is difficult to find oligotrophic lakes; therefore, it is difficult to observe a normal distribution of zooplankton biodiversity along a trophic gradient.

The zooplankton response to the depth and mixing regime is complex due to the interplay of environmental processes (such as eutrophication) and certain traits of micro-invertebrates. Thus, the present study focused on how depth and mixing regime affect zooplankton diversity in lakes. The authors also investigated the vertical distribution of diversity across a transparency gradient (used as a trophy proxy) among the lakes. We hypothesized that the epilimnion of dimictic lakes is less diverse than that of polymictic lakes. Furthermore, we hypothesized that when the transparency decreases, the biodiversity of zooplankton decreases and that the zooplankton composition would be more similar among lakes of the same mixing type.

**Figure 1:**
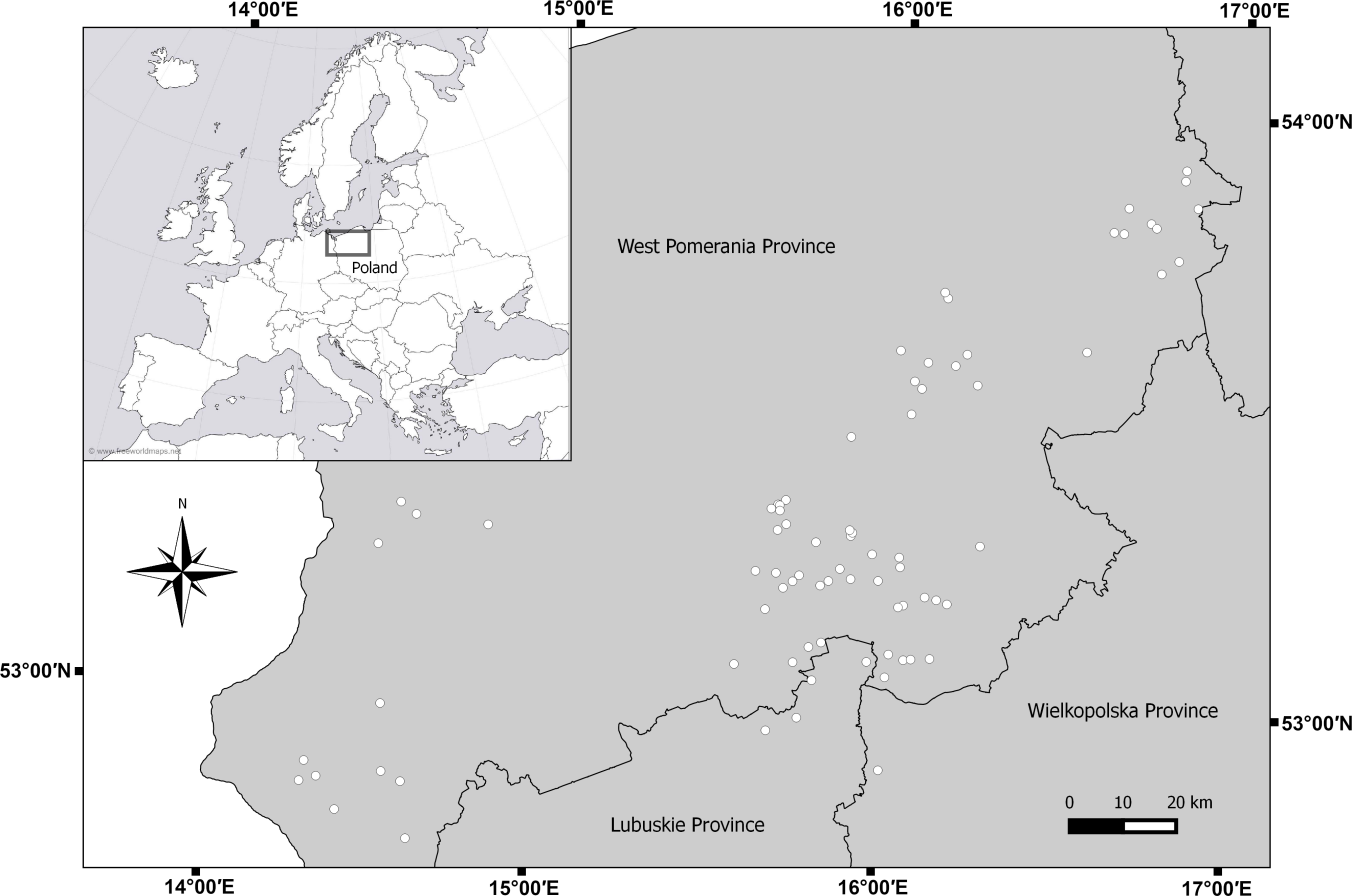
Map of sampled lakes in north-west Poland.

**Table 1:** Characteristics of the study lakes of northwestern Poland.

Lake		Coordinates	Maximum depth	Surface	Volume	Mictic type	Secchi disc depth	TSI	Number of samples		
Number	Name	ETRS89	(m)	(ha)	(k m^−3^)	(P-Polymitic D-Dimictic)	(m)	Trophic state index (SD)	Epilimnion	Metalimnion	Hypolimnion
1	Adamowo	53°12′40.3″N 15°44′58.6″E	34.4	112	8,137	D	1.6	53.2	3	2	1
2	Baba	53°20′52.2″N 15°43′13.5″E	2.5	3	45	P	1.3	56.2	1	–	–
3	Białe	53°49′07.6″N 16°39′03.6″E	19	31.3	2,391	D	3.4	42.4	1	1	1
4	Bielińskie	52°50′22.7″N 14°26′41.0″E	9	27.5	1,098	P	1.8	51.9	2	–	–
5	Bielskie	53°51′53.5″N 16°51′37.5″E	23	257.9	15,977	D	0.8	63.2	3	3	2
6	Binowskie	53°18′36.9″N 14°38′11.0″E	9.4	52.4	2,905	D	5,0	36.8	2	1	–
7	Bobrowo Duże	53°17′30.3″N 15°54′27.6″E	34.1	25.2	3,132	D	4.0	40.0	2	2	2
8	Bobrowo Małe	53°17′50.3″N 15°54′43.1″E	10.1	11.6	542	P	3.5	41.9	2	–	–
9	Bytyń	53°16′58.5″N 16°16′23.2″E	41	877.1	91,535	D	2.6	46.4	4	4	4
10	Chełm Dolny	52°51′15.5″N 14°37′29.8″E	5	17.5	524	P	1.6	53.7	2	–	–
11	Chomętowo	53°12′21.6″N 15°49′39.3″E	2.8	12	180	P	0.8	63.2	2	–	–
12	Cieszęcino	53°55′41.7″N 16°49′29.6″E	38	102.2	13,790	D	4.0	40.0	2	2	1
13	Czaplino	53°33′15.8″N 16°14′57.5″E	22.9	108.3	13,345	D	3.0	44.4	2	2	1
14	Czarne	53°13′26.8″N 15°42′05.8″E	2.8	7.7	116	P	0.4	75.1	2	–	–
15	Damskie	53°50′13.8″N 16°43′40.0″E	14.9	68	2,791	D	4.2	39.3	2	1	1
16	Długie	53°41′57.7″N 16°09′21.0″E	4.7	15	374	P	2.0	50.0	1	–	–
17	Dołgie	53°46′29.5″N 16°48′37.0″E	19.8	310	20,672	D	2.0	50.3	3	2	1
18	Dominikowo	53°12′51.5″N 15°51′00.9″E	16.5	78.6	7,327	D	3.5	41.9	3	3	2
19	Drawsko	53°35′09.3″N 16°11′06.0″E	82.2	1871.5	331,443	D	3.1	43.9	4	4	4
20	Dubie	53°05′40.5″N 16°01′38.1″E	5.1	19.2	374	P	1.9	50.8	2	–	–
21	Generalskie	53°20′23.0″N 15°41′56.3″E	3	6	108	P	1.7	52.4	1	–	–
22	Glinne	53°17′28.3″N 14°40′54.1″E	16.4	75.6	6,239	D	2.4	47.4	2	1	1
23	Grażyna	53°13′18.1″N 15°46′01.8″E	5	64	2,432	P	2.1	49.7	2	–	–
24	Jaworze	53°20′18.1″N 15°42′13.8″E	3.5	18	360	P	1.3	56.2	2	–	–
25	Karpino	53°10′55.7″N 15°41′19.7″E	2.5	28.5	1,138	P	1.3	56.2	2	–	–
26	Korytnica	53°13′04.9″N 15°59′23.8″E	4.7	111.3	2,637	P	0.5	69.1	3	–	–
27	Kosino	53°04′01.2′N 15°35′47′E	12	50	2,500	D	1.6	53.7	2	1	1
28	Krosino	53°33′25.9″N 16°04′14.6″E	17.2	177.2	12,734	D	2.0	50.0	2	1	1
29	Królewskie	52°53′54.8″N 16°00′41.4″E	3.4	48.8	875	P	0.6	68.6	2	–	–
30	Krzywe	53°42′33.0″N 16°08′47.3″E	5	13.5	405	P	2.0	50.0	1	–	–
31	Krzywe Dębsko	53°14′07.6″N 15°52′51.4″E	18.1	121.6	7,132	D	3.7	41.3	3	1	1
32	Krzywy Róg	53°11′57.8″N 15°43′24.5″E	5	19.8	287	P	0.8	63.2	2	–	–
33	Lipowo	52°44′37.0″N 14°42′13.5″E	10.3	12.5	563	P	1.1	58.6	2	–	–
34	Liptowskie	53°10′59.1″N 16°11′10.1″E	29	134.9	12,457	D	2.0	50.0	3	2	2
35	Lubicz	53°15′32.0″N 16°02′48.5″E	7	33.4	1,369	D	1.0	60.7	2	1	1
36	Lubicz Mały	53°14′33.0″N 16°03′01.0″E	1.9	11.6	108	P	2.0	50.0	2	–	–
37	Lubie	53°27′32.6″N 15°53′50.2″E	46.2	1439	169,881	D	3.0	44.2	3	3	3
38	Łabędzin	52°49′49.2″N 14°23′52.8″E	5	12.5	374	P	1.6	53.2	1	–	–
39	Łobez	53°54′38.8″N 16°49′21.1″E	17	45.5	2,564	D	3.1	43.7	1	1	1
40	Marta	53°10′41.5″N 16°03′48.1″E	25	66.1	5,111	D	6.0	34.1	3	2	2
41	Martwe	53°49′13.7″N 16°37′20.6″E	2	3.8	57	P	0.2	83.2	2	–	–
42	Mąkowarskie	53°16′43.7″N 15°48′37.5″E	31.2	170.5	23,197	D	2.0	50.0	3	2	2
43	Miedwie	53°16′49.9″N 14°53′06.2″E	43.8	3527	681,672	D	1,6	53,7	3	2	2
44	Mieszkowickie	52°47′07.4″N 14°30′07.4″E	1	7	49	P	0.6	67.4	2	–	–
45	Młyńskie	53°05′23.6″N 16°08′36.4″E	6	38	898	P	1.5	54.2	2	–	–
46	Młyńskie Kalisz	53°18′05.7″N 15°54′14.6″E	15.3	14.8	831	D	1.8	51.5	2	2	2
47	Morzycko	52°51′53.1″N 14°24′30.5″E	60.7	342.7	49,827	D	1.0	60.2	4	1	1
48	Osiek	52°57′27.6″N 15°41′32.3″E	35.3	538.9	50,065	D	3.5	41.8	4	3	2
49	Ostrowiec	53°04′50.2″N 15°57′59.5″E	28.5	387.6	36,433	D	2.1	49.5	3	2	2
50	Palowskie	53°19′46.4″N 15°42′17.5″E	3	10	180	P	1.4	55.2	2	–	–
51	Pańskie	53°17′47.4″N 15°42′02.9″E	4	46	690	P	1.0	60.0	3	–	–
52	Piaski	53°06′05.3″N 15°48′08.7″E	15.7	77.7	6,832	D	6.1	33.9	2	2	1
53	Płociowe	53°10′29.1″N 16°02′56.8″E	25	35.3	3,620	D	7.0	32.0	2	2	1
54	Promień	52°50′20.9″N 14°40′49.8″E	4.4	36	720	P	1.0	60.7	2	–	–
55	Radęcino	53°02′44.5″N 15°48′54.7″E	15	174.4	7,341	D	2.1	49.7	4	2	1
56	Rakowe	53°06′35.8″N 15°50′13.2″E	15	49.9	3,216	D	2.2	49.0	2	2	1
57	Rybnica	53°13′33.0″N 15°38′37.4″E	2.9	12	180	P	0.7	65.7	2	–	–
58	Rzepowo	53°35′22.7″N 16°06′24.6″E	5.2	38.9	1,016	P	0.9	61.1	2	–	–
59	Siecino	53°36′29.5″N 16°01′38.9″E	44.2	729	104,442	D	6.3	33.6	3	3	3
60	Stepieńskie	53°49′46.7″N 16°44′37.4″E	33.1	36.7	3,193	D	3.0	44.2	2	1	1
61	Szczuczarz	53°03′20.8″N 16°01′09.2″E	17.4	138.2	8,708	D	4.5	38.3	3	1	1
62	Szerokie	53°13′08.7″N 15°54′44.6″E	15.8	76.3	4,680	D	4.1	39.7	2	1	1
63	Śmiadowo	53°37′00.8″N 16°33′23.1″E	15	129.9	7,498	D	2.7	46.0	2	1	1
64	Trzebuń	53°18′25.1″N 15°43′28.4″E	20	136.2	12,591	D	3.5	41.8	4	3	3
65	Trzygłowskie	52°58′07.5″N 14°36′42.1″E	5.6	43.6	1,350	P	0.6	67.4	2	–	–
66	Tuczno	53°11′35.7″N 16°07′22.2″E	20.2	128.9	11,669	D	1.8	51.5	3	1	1
67	Wąsosze	53°30′04.9″N 16°03′54.0″E	8.5	326.4	11,330	P	0.6	67.8	3	–	–
68	Wełtyń	53°14′16.0″N 14°34′45.2″E	11.6	310.1	13,971	P	3.9	40.4	3	–	–
69	Wielgie	52°58′53.0″N 15°46′38.5″E	6.8	136.9	3,078	P	0.7	64.5	3	–	–
70	Wielimie	53°45′10.5″N 16°45′41.8″E	5.5	1754.6	40,129	P	0.3	75.8	3	–	–
71	Wieliż	53°15′44.5″N 15°58′12.5″E	4.2	31.1	832	P	1.0	60.0	2	–	–
72	Wierzchowo	53°51′43.7″N 16°39′46.4″E	26.5	731	70,213	D	3.0	44.2	3	2	1
73	Wilcze	53°19′55.9″N 15°40′49.8″E	2.5	4.6	60	P	1.8	51.5	2	–	–
74	Wilczkowo	53°32′42.2″N 16°05′30.5″E	26.7	300.4	23,301	D	2.0	50.0	2	2	1
75	Wyrwy Wielkie	53°04′28.9″N 15°45′35.6″E	12.5	227.6	10,362	D	1.4	55.4	4	1	1
76	Załom	53°05′09.6″N 16°04′10.0″E	21.5	104.7	5,657	D	1.9	51.0	3	1	1
77	Zamkowe	53°11′21.7″N 16°09′20.6″E	9.5	17	715	P	2.0	50.0	1	–	–
78	Zatom Mały	53°05′15.3″N 16°05′25.3″E	2.7	22.9	312	P	1.9	50.8	1	–	–
79	Żerdno	53°36′21.2″N 16°12′59.7″E	36	205	31,240	D	3.1	43.9	2	2	2

## Methods

A set of zooplankton samples was collected once for each lake during the day in the summer, from 15 July 2011 to 15 August A total of 329 zooplankton samples were collected from 79 lakes in northwestern Poland (Fig. 1). Lakes were chosen according to depth gradient (Choiński, 2006). All sampled lakes are affected by commercial fishing and none of the lakes are temporary. Completely stratified lakes and lakes that were deep enough to partially stratify (lakes with a thermocline) were classified dimictic (deep lakes). Lakes without a fully developed thermocline were classified polymictic (shallow lakes). According to those criteria, we classified 36 polymictic and 43 dimictic lakes (Table 1). Sampling stations were set up at the deepest point in the lake (Jańczak, 1996). If the lake was morphologically diverse, several sampling stations were established. Individual thermal layers were determined for each sampling station in each lake, based on temperature measurements at 1-m intervals. In the case of shallow lakes, the sampling site was located at the furthest distance from plants and the littoral zone. In thermally stratified lakes, samples were taken from three zones: the epilimnion, the metalimnion, and the hypolimnion, whereas in shallow lakes, the samples were collected only from the epilimnion (Lewis Jr, 1983).

The summer period was chosen based on the methodology used by other authors in zooplankton research (Karabin, 1985; Dodson et al., 2009). Summer stagnation results in the accumulation of factors (e.g., color, chlorophyll and phosphorus concentration) affecting the trophic state of lakes (Karabin, 1985). During the stagnation period, the abiotic and biotic factors fluctuate very little, compared to the spring and autumn periods, when intensive water mixing occurs. Table 2 shows the ranges of temperature, dissolved oxygen, pH and conductance that were measured with a Hydrolab DS5 sensor (USA).

**Table 2:** Values of basic physicochemical variables of lakes. The ranges of temperature, dissolved oxygen, pH and conductance from thermal layers.

	Epilimnion	Metalimnion	Hypolimnion
Temperature (°C)	18.9–25.0	12.0–17.0	4.0–11.0
O_2_ (mg dm^−3^)	2.4–13	0.3–11.0	0–9.7
pH	4.45–9.75	6.4–8.9	4.4–8.4
Conductance (µS cm^−2^)	53–730	59–537	65–544

For water samples, a Van Dorn 3-liter sampler was used. 50 liters of water was taken at each sampling site and from each layer and passed through a plankton mesh of 30 µm. The samples from each zone were not combined with other thermal zones. Concentrated samples were poured into a 110-ml tube and fixed in a 4% formalin solution. Zooplankton was analyzed in four subsamples in 2-ml plankton chambers using a Nikon Eclipse 50i microscope and a Zeiss Primo Vert reverse microscope. Zooplankton samples were identified using taxonomic keys (e.g., Einsle, 1996; Hołyńska et al., 2003; Radwan, 2004; Benzie, 2005; Błędzki & Rybak, 2016).

Zooplankton biodiversity was calculated with two commonly used indicators: species richness SR (number of species) and the Shannon index SI (log base e) (Shannon & Weaver, 1949). Species richness is the simplest and fundamental measurement of community. It is also the basis of many ecological models of community structure (May, 1988). Maintaining biodiversity is also a central objective for various monitoring and management projects (Williams & Gaston, 1994; Green et al., 2005; Pereira & Cooper, 2006). Another commonly used biodiversity metric is the Shannon index. Entropy is a reasonable index of diversity, which takes SR and species evenness into consideration (Shannon & Weaver, 1949; Jost, 2006).

An index based on the visibility of the Secchi disc (SD) was used to determine the trophic state of the lakes (TSI_SD_) (Carlson, 1977). The TSI_SD_ was calculated using the 60-14.41 ln(SD) formula, where SD is transparency in meters. The TSI_SD_ value referred to all thermal zones in a given lake. The SD is affected by various water quality parameters, such as chlorophyll, total phosphorus concentration, and color in addition to light scattering and light absorption (Carlson, 1977; Fee et al., 1996; Brezonik et al., 2015). Despite the numerous variables affecting transparency, the TSI_SD_ is commonly used as a key eutrophication indicator of different water body types (Karabin, 1985; Pyhälä, Fleming-Lehtinen & Laamanen, 2014; Stock, 2015; Binding et al., 2015; Heddam, 2016; Alikas & Kratzer, 2017). Therefore, the Secchi depth (SD) is an important visual indicator of water clarity that can be used for the water quality index calculation (Carlson, 1977; Alexakis et al., 2016; Heddam, 2016; Ochocka & Pasztaleniec, 2016).

To determine the correlations and significant differences in zooplankton biodiversity, we used the mean value for SR and SI for lakes and thermal layers (Statistica 12 software; StatSoft, Tulsa, OK, USA). To find the best predictors for zooplankton biodiversity a multiple stepwise regression was used (*P* < 0.05) (Sokal & Rohlf, 1995). The relationship between TSI_SD_ and depth was verified by the Pearson correlation (*P* < 0.05). The parametric one-way ANOVA and the post hoc Duncan test were used to determine significant differences in zooplankton biodiversity between mictic lake types and thermal layers (*P* < 0.05). The rarefaction method and the “EstimateS” software were used to calculate the species number (SR) and the Shannon index (SI) values. These tools were used to test differences among lakes types and thermal layers. In an effort to avoid bias for lakes with larger sample numbers, we followed the rarefaction methods, and used all samples for each layer. Recent examples emphasize the importance of quantifying SR using taxon sampling curves (Allen et al., 1999; Gotelli & Colwell, 2001; Chao et al., 2014). Therefore, we based our calculations on the number of samples. Based on Sørensen similarity analyses (MVSP 3.22 software), we developed four levels of taxonomic similarity (<0.5—low degree of similarity; 0.5–0.65—moderate degree of similarity; 0.8–0.65—high degree of similarity; >0.8—very high degree of similarity) and compared similarities between pairs (i.e., between two lakes) for polymictic and dimictic lakes, and their percentage contribution to the similarity classification. Nonmetric multidimensional scaling (NMDS) (Bray–Curtis distance metric, a square root transformation, and Wisconsin double standardization) describe the similarity patterns in species composition among lakes in terms of mictic type, thermal layer and the TSI_SD_ value.

## Results

The ranges of environmental variables in the 36 polymictic and 43 dimictic lakes are shown in Tables 1 and 2. Lake depth was significantly correlated with the TSI_SD_ value (*R* =  − 0.44; *p* < 0.001). We identified a total of 151 taxa in the sampled lakes (Table 3). The most frequent taxa (with the frequency of occurrence of more than 75% among lakes) were: *Keratella cochlearis* 100%, *Polyarthra vulgaris* 94%, *Mesocyclops leuckarti* 88%, *Trichocerca similis* 82%, *Daphnia cucullata* 84%, *Diaphanosoma brachyurum* 78%, *K. cochlearis v. tecta* 77%, and *Thermocyclops oithonoides* 75%.

**Table 3:** Frequency (%) of zooplankton taxa in examined lakes.

	Lakes	Epilimnion		Metalimnion	Hypolimnion
	*n* = 79	Polimictic (*n* = 36)	Dimictic (*n* = 43)	*n* = 43	*n* = 42
Rotifera					
*Anuraeopsis fissa*	42	56	21	23	10
*Ascomorpha ecaudis*	3	3	2	0	0
*Ascomorpha ovalis*	51	28	65	35	13
*Ascomorpha saltans*	52	61	42	21	3
*Ascomorphella volvocicola*	4	0	7	0	0
*Asplanchna priodonta*	73	64	72	56	18
*Asplanchna sieboldi*	1	3	0	0	0
Bdelloidea	37	33	30	14	8
*Brachionus angularis*	33	47	14	7	10
*Brachionus calyciflorus*	9	11	5	0	3
*Brachionus diversicornis*	15	28	5	2	0
*Brachionus quadridentatus*	4	8	0	0	0
*Brachionus urceolaris*	1	0	2	0	3
*Cephalodella gibba*	1	3	0	0	0
*Collotheca mutabilis*	35	36	33	12	0
*Colurella adriatica*	3	6	0	0	0
*Colurella colurus*	6	6	7	0	0
*Colurella obtusa*	5	6	5	0	0
*Colurella uncinata*	8	14	2	0	0
*Conochillus unicornis*	58	44	60	37	8
*Conochilus hippocrepis*	5	0	5	9	0
*Euchlanis deflexa deflexa*	6	6	7	0	0
*Euchlanis dilatata dilatata*	41	39	42	9	3
*Euchlanis meneta*	1	3	0	0	0
*Filinia longiseta*	53	56	21	28	20
*Filinia terminalis*	9	0	0	5	13
*Gastropus hyptopus*	4	3	2	5	3
*Gastropus stylifer*	62	53	63	44	23
*Hexarthra mira*	4	6	2	0	0
*Keratella cochlearis v. cochlearis*	100	100	98	100	90
*K. cochlearis v. hispida*	77	72	63	72	45
*K. cochlearis v. macracantha*	45	31	42	31	11
*K. cochlearis v. tecta*	77	92	63	51	38
*Keratella hiemalis*	8	0	0	9	13
*Keratella quadrata*	68	44	35	74	43
*Kellicottia longispina*	66	31	81	86	65
*Lecane bulla*	9	11	5	2	0
*Lecane closterocerca*	25	28	12	7	13
*Lecane depressa*	1	3	0	0	0
*Lecane flexilis*	1	3	0	0	0
*Lecane hamata*	8	11	2	2	3
*Lecane ludwigii*	4	6	2	0	0
*Lecane luna*	13	6	19	0	3
*Lecane lunaris*	11	3	19	2	0
*Lecane perpusilla*	1	3	0	0	0
*Lecane scutata*	3	3	2	0	0
*Lecane tenuiseta*	1	0	0	2	0
*Lepadella acuminata*	1	3	0	0	0
*Lepadella ovalis*	10	6	12	2	0
*Lepadella quadricarinata*	3	6	0	0	0
*Lepadella rhomboides*	1	3	0	0	0
*Monommata maculata*	3	3	2	0	0
*Mytilina mucronata*	1	3	0	0	0
*Notholca labis*	1	0	0	2	0
*Notholca squamula*	1	0	0	0	3
*Plationus patulus*	3	3	2	0	0
*Platyias quadricornis*	3	3	2	0	0
*Ploesoma hudsoni*	9	8	5	7	0
*Polyarthra dolichoptera*	3	0	0	6	0
*Polyarthra eryptera*	59	61	53	21	3
*Polyarthra longiremis*	15	6	23	7	5
*Polyarthra major*	23	8	33	12	3
*Polyarthra minor*	1	3	0	0	0
*Polyarthra remata*	52	56	37	19	8
*Polyarthra vulgaris*	95	89	98	56	45
*Pompholyx sulcata*	70	67	63	63	48
*Scaridium longicaudum*	1	3	0	0	0
*Synchaeta kitina*	38	31	42	14	10
*Synchaeta lakowitziana*	1	0	0	0	3
*Synchaeta oblonga*	4	6	2	2	0
*Synchaeta pectinata*	13	17	7	0	3
*Synchaeta stylata*	1	0	0	0	0
*Synchaeta tremula*	1	3	0	0	0
*Testudinella patina*	5	8	2	0	0
*Trichocerca capucina*	72	72	60	44	18
*Trichocerca cylindrica*	6	8	5	0	0
*Trichocerca elongata*	4	6	2	0	0
*Trichocerca insignis*	4	6	2	0	0
*Trichocerca porcellus*	1	0	0	0	0
*Trichocerca pusilla*	42	61	26	7	10
*Trichocerca rattus*	4	8	0	0	0
*Trichocerca rousseleti*	49	36	56	30	13
*Trichocerca similis*	82	81	81	65	35
*Trichocerca simonei*	3	0	5	2	0
*Trichocerca stylata*	4	8	0	0	0
*Trichotria pocillum*	4	0	5	2	0
Cladocera					
*Alona costata*	4	8	0	0	0
*Alona intermedia*	3	0	5	0	0
*Alona rectangula*	6	8	5	0	0
*Alonella excisa*	1	0	2	0	0
*Alonella exigua*	1	3	0	0	0
*Alonella nana*	18	22	14	0	0
*Bosmina longirostris*	56	53	42	28	28
*Bythotrephes longimanus*	5	0	0	9	0
*Ceriodaphnia pulchella*	9	11	7	0	3
*Ceriodaphnia quadrangula*	68	72	51	33	20
*Chydorus gibbus*	3	0	5	0	3
*Chydorus latus*	4	3	5	0	0
*Chydorus ovalis*	3	3	5	0	0
*Chydorus sphaericus*	46	47	42	26	10
*Daphnia cucullata*	85	75	93	91	60
*Daphnia galeata*	9	6	5	12	13
*Daphnia hyalina*	13	0	9	21	15
*Daphnia longiremis*	1	0	0	0	3
*Daphnia longispina*	46	8	40	70	35
*Diaphanosoma brachyurum*	78	61	86	77	45
*Disparalona rostrata*	5	6	5	0	0
*Eubosmina coregoni*	68	50	79	67	58
*Eubosmina gibbera*	3	3	2	0	0
*Eubosmina l. kessleri*	1	0	0	2	0
*Eubosmina longicornis*	1	0	2	0	0
*Eubosmina longispina*	3	0	0	5	3
*Eubosmina thersites*	1	3	0	0	0
*Holopedium gibberum*	1	0	0	2	0
*Latona setifera*	1	3	0	0	0
*Leptodora kindtii*	37	8	19	56	13
*Pleuroxus aduncus*	1	3	0	0	0
*Pleuroxus striatus*	1	0	2	0	0
*Pleuroxus trigonellus*	3	6	0	0	0
*Pleuroxus truncatus*	3	6	0	0	0
*Pleuroxus uncinatus*	1	0	2	0	0
*Polyphemus pediculus*	8	14	2	0	0
*Pseudochydorus globosus*	1	0	2	0	0
*Scapholeberis mucronata*	9	17	0	2	0
*Sida crystallina*	10	11	9	0	0
*Simocephalus exspinosus*	1	0	2	0	0
*Simocephalus serrulatus*	1	3	0	0	0
Copepoda					
*Acanthocyclops robustus*	3	6	0	0	0
*Cryptocyclops bicolor*	3	3	2	0	0
*Cyclops abyssorum*	20	0	2	26	20
*Cyclops kolensis*	4	0	0	0	8
*Cyclops scutifer*	4	0	0	7	5
*Cyclops vicinus*	4	3	0	5	3
*Diacyclops bicuspidatus*	27	0	0	35	28
*Diacyclops bisetosus*	1	0	0	2	3
*Eucyclops macruroides*	5	3	5	0	3
*Eucyclops macrurus*	1	0	2	0	0
*Eucyclops serrulatus*	5	3	0	7	0
*Eucyclops speratus*	1	0	0	2	0
*Eudiaptomus gracilis*	34	11	44	51	33
*Eudiaptomus graciloides*	51	25	58	65	53
*Eurytemora lacustris*	6	0	0	9	13
*Heterocope appendiculata*	4	0	2	7	3
*Macrocyclops albidus*	1	3	0	0	0
*Megacyclops viridis*	4	0	2	2	3
*Mesocyclops leuckarti*	89	78	91	86	35
*Microcyclops varicans*	3	0	5	0	0
*Paracyclops fimbriatus*	1	0	2	0	0
*Paracyclops poppei*	1	0	2	0	0
*Thermocyclops crassus*	39	31	21	37	13
*Thermocyclops oithonoides*	76	67	79	81	58
naupli Cyclopoida	100	100	100	81	75
naupli Calanoida	82	61	100	79	53
Kopepodit Cyclopoida	97	94	100	93	78
Kopepodit Calanoida	76	47	98	81	55

**Figure 2:**
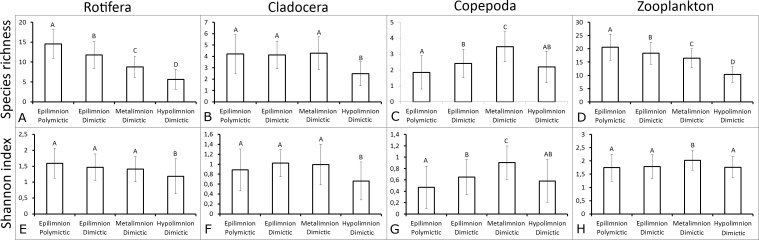
Mean ± SD values of species richness and Shannon biodiversity index for polymictic and dimictic lakes (with their thermal layers). (A) Rotifera SR. (B) Cladocera SR. (C) Copepoda SR. (D) Zooplankton SR. (E) Rotifera SI. (F) Cladocera SI. (G) Copepoda SI. (H) Zooplankton SI. Epilimnion of polymictic lakes (*n* = 36), epilimnion of dimictic lakes (*n* = 43), metalimnion (*n* = 43) and hypolimnion (*n* = 42). Different letters indicate significant differences (*p* < 0.05) in values of variables.

Multiple regression revealed that the TSI_SD_ and depth significantly affected the Rotifera SR, Copepoda SR, and Copepoda SI. However, the TSI_SD_ significantly affected the total zooplankton SR (*P* < 0.05) (Table 4). The analysis (*R* value) explained 25%–38% of the variability in the SR of Rotifera, Copepoda and total zooplankton and the SI of Copepoda (*P* < 0.05). The Duncan test results show that the mictic type had a significant effect on the zooplankton biodiversity (Fig. 2). Rotifera SR was significantly higher in polymictic lakes than in dimictic lakes and significantly affected by thermal zone (*p* < 0.05). The deepest layers of dimictic lakes presented the lowest levels of Cladocera SR and SI (*p* < 0.05). The Copepoda SR and SI values in the metalimnion of dimictic lakes were significantly higher than in the epilimnion of both shallow and deep lakes (*p* < 0.05).

**Table 4:** Significances of the effects of depth and TSI_SD_ on the SR and SI of zooplankton based on multiple regression. Diversity indices for which none of the predictors were significant are not reported.

	Rotifera SR	Copepoda SR	Zooplankton SR	Copepoda SI
TSI_SD_ (*β*)	0.17^*^	−0.19^*^	0.06	−0.16^*^
Depth (*β*)	−0.28^***^	0.19^*^	−0.22^**^	0.17^*^
*R*	0.38	0.31	0.25	0.28
*F*(2.161)	13.34	8.62	5.46	6.74
*p*	0.0000	0.0003	0.0051	0.0015

**Notes.**

* *P* < 0.05.

** *P* < 0.01.

*** *P* < 0.001.

**Figure 3:**
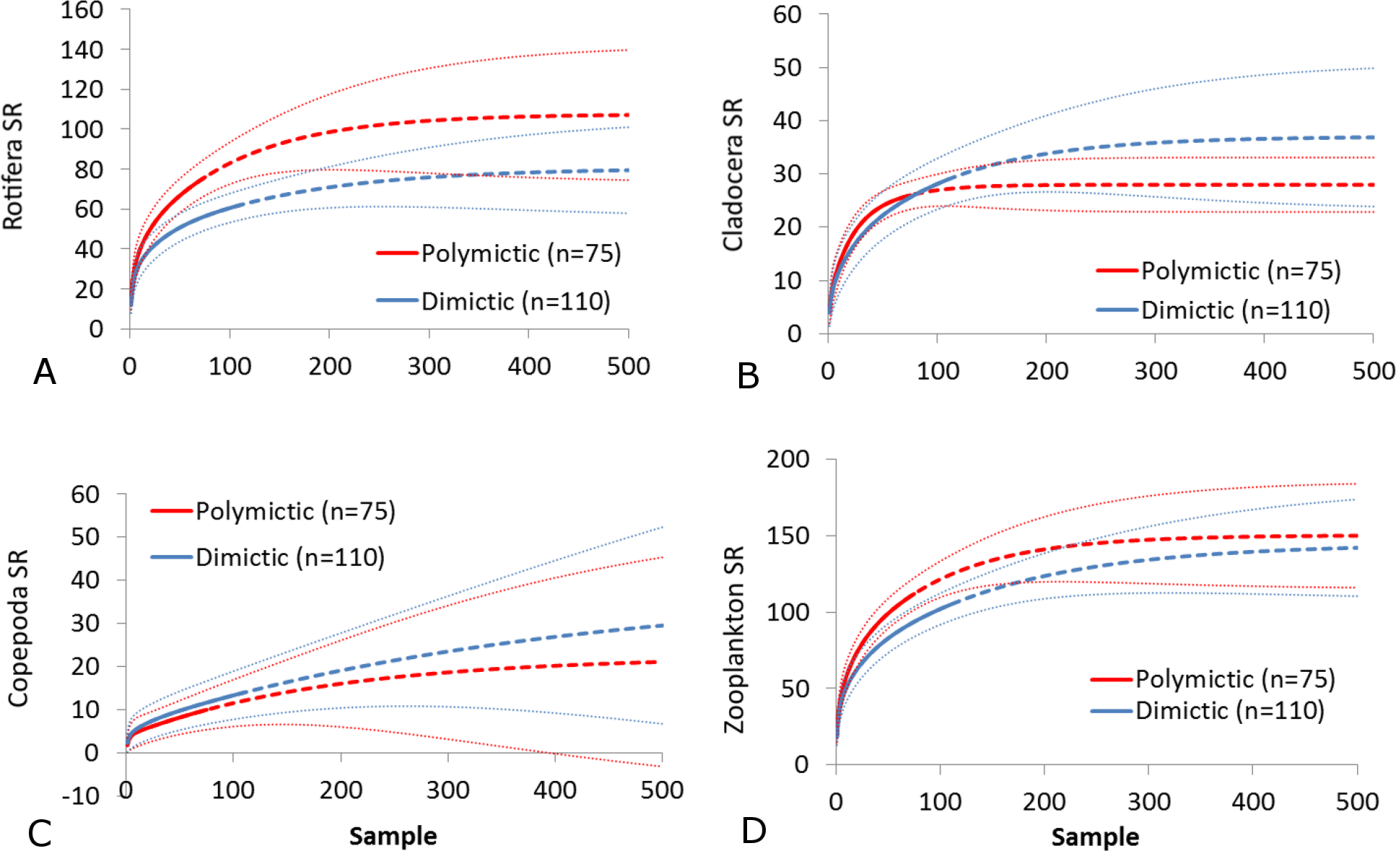
Zooplankton species richness in lakes with different mictic types (epilimnion only). (A) Rotifera. (B) Cladocera. (C) Copepoda. (D) Zooplankton. Sample-based rarefaction (solid line) and extrapolation curves (dotted line). Thin dotted line—a 95% confidence interval.

**Figure 4:**
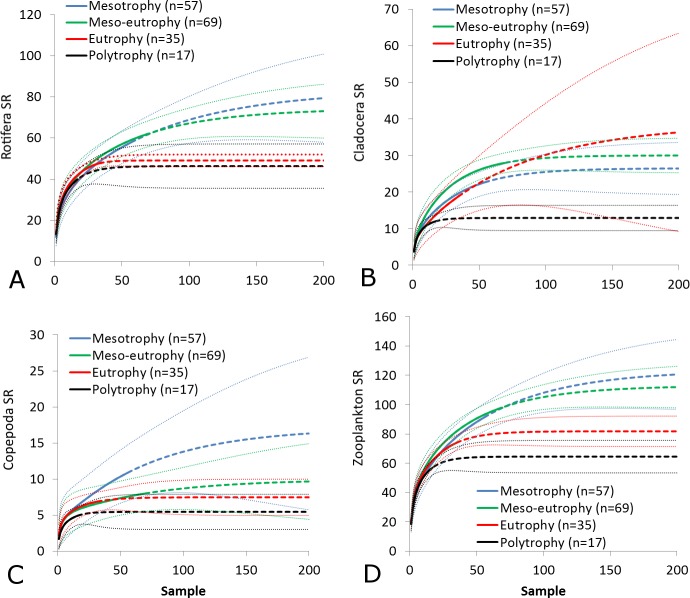
Zooplankton species richness in the epilimnion with a different trophic status. (A) Rotifera. (B) Cladocera. (C) Copepoda. (D) Zooplankton. Sample-based rarefaction (solid line) and extrapolation curves (dotted line). Thin dotted line—a 95% confidence interval.

**Figure 5:**
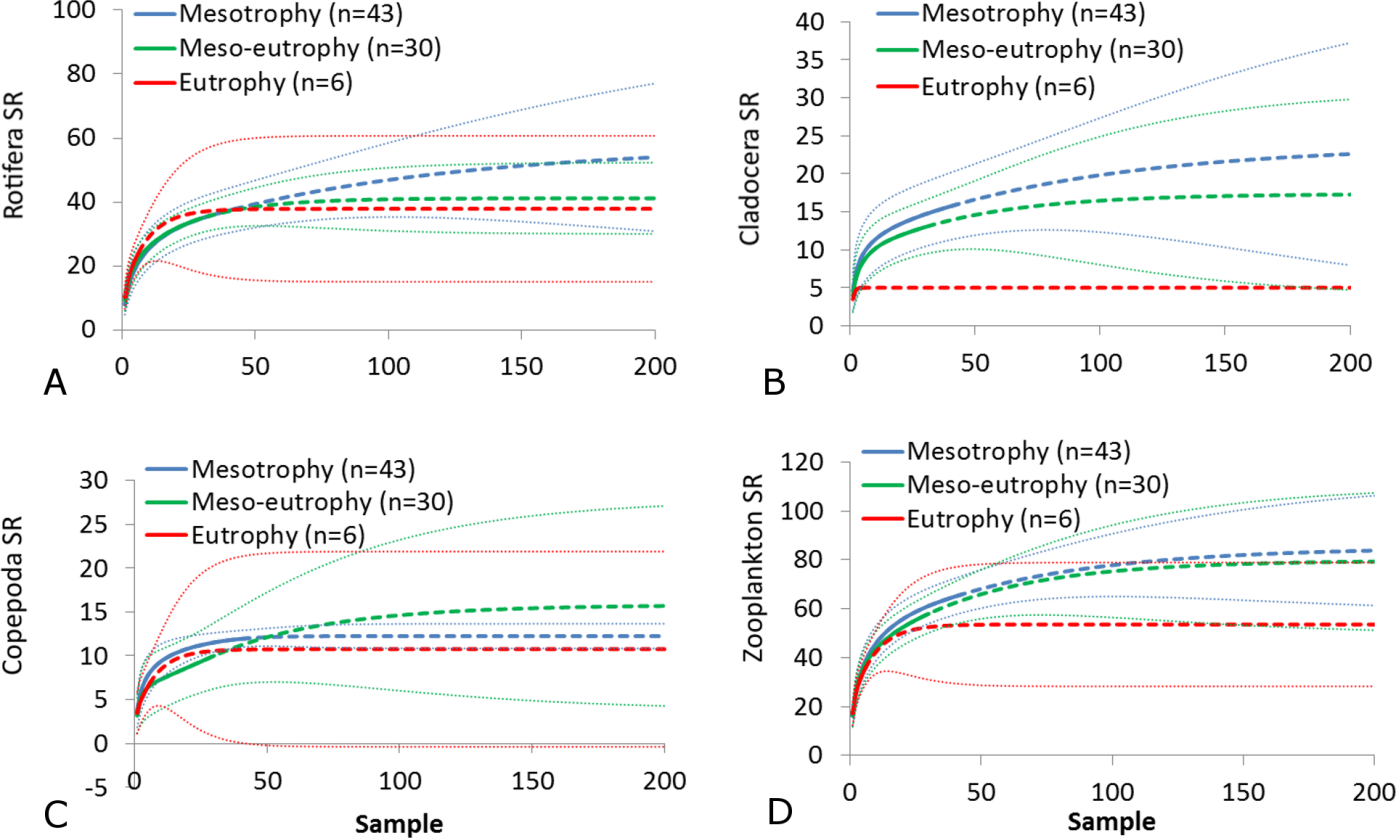
Zooplankton species richness in the metalimnion with a different trophic status. (A) Rotifera. (B) Cladocera. (C) Copepoda. (D) Zooplankton. Sample-based rarefaction (solid line) and extrapolation curves (dotted line). Thin dotted line—a 95% confidence interval.

**Figure 6:**
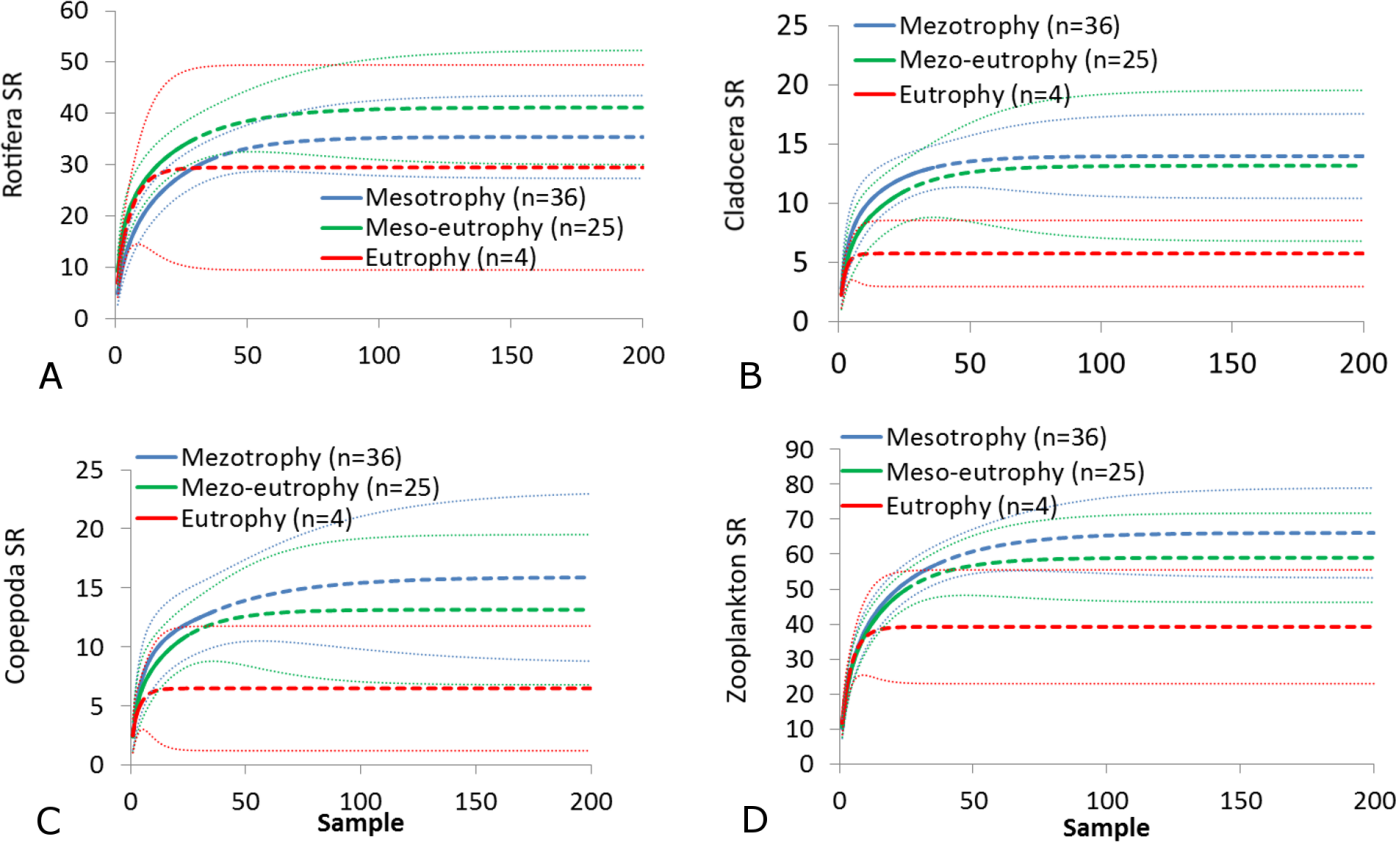
Zooplankton species richness in the hypolimnion with a different trophic status. (A) Rotifera. (B) Cladocera. (C) Copepoda. (D) Zooplankton. Sample-based rarefaction (solid line) and extrapolation curves (dotted line). Thin dotted line—a 95% confidence interval.

**Figure 7:**
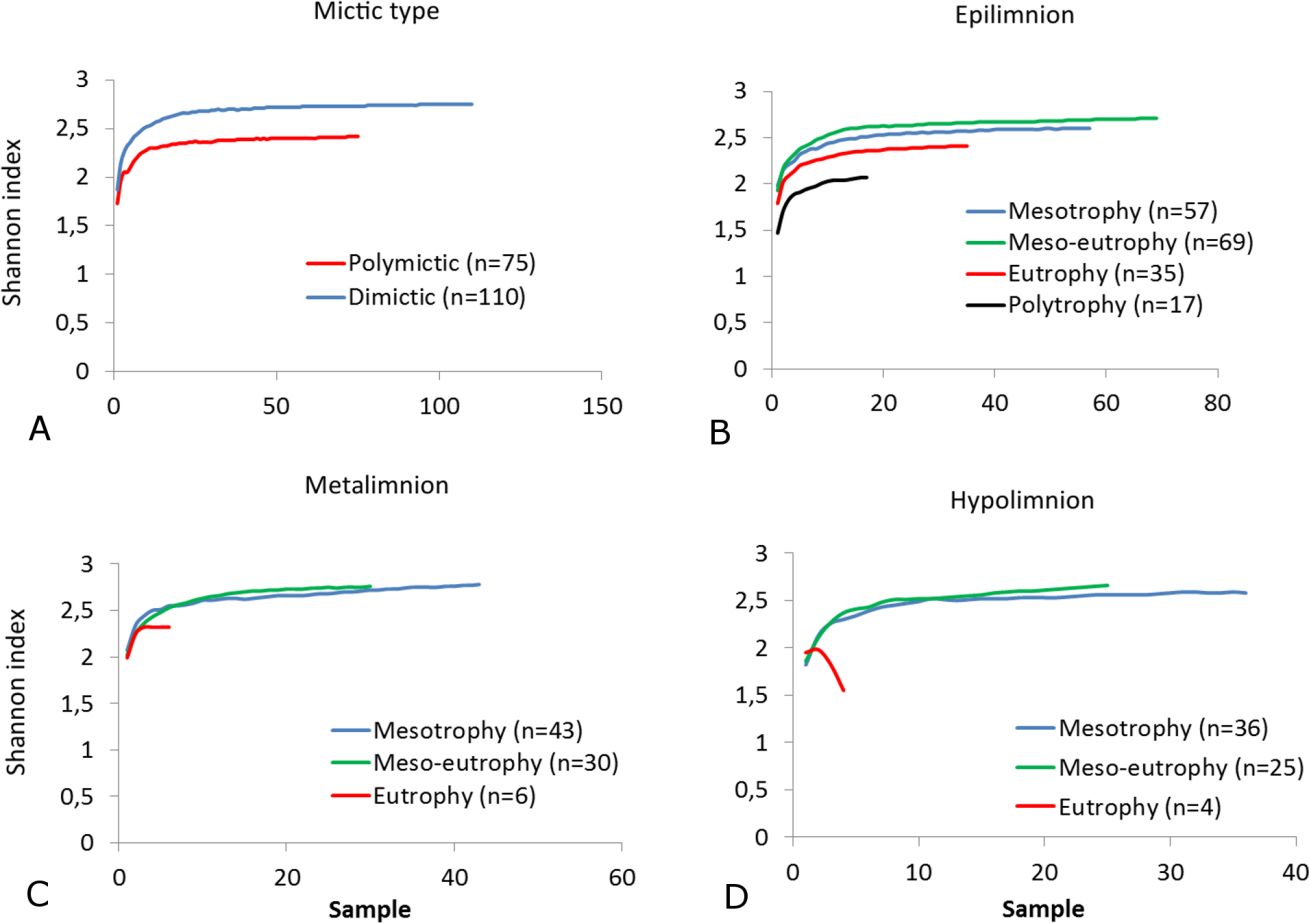
Shannon biodiversity of zooplankton in lakes with a different mictic type and thermal layers. (A) Mictic types. (B) Epilimnion. (C) Metalimnion. (D) Hypolimnion. Sample-based rarefaction (solid line).

The SR rarefaction curve of zooplankton showed differences between the groups of polymictic vs. dimictic lakes (Fig. 3), and between lakes with a different trophic state (Fig. 4). In general, the SR of zooplankton in the epilimnion showed a higher value in lakes with a low trophic state. The SR of Rotifera and Copepoda was the highest in the mesotrophic lakes, and the lowest in the polytrophic lakes. In contrast, in the above results, the highest Cladocera SR was found in the eutrophic lakes, whereas fewer species were present in the polytrophic lakes. In all cases, polytrophic waters represented the lowest SR of the organisms studied. In the metalimnion, the SR of Rotifera and especially of Cladocera, was the highest in the mesotrophic lakes, and lowest in the eutrophic lakes (Fig. 5). Furthermore, the highest Copepoda SR was found in the mesoeutrophic lakes, whereas fewer species were present in the eutrophic lakes. In all cases, eutrophic waters represented the lowest SR. In the hypolimnion, the Crustacea SR was the highest in the mesotrophic lakes and the lowest in the eutrophic lakes (Fig. 6). The highest Rotifera SR value was recorded in the mesoeutrophic lakes, whereas fewer species were present in the eutrophic lakes. Eutrophic waters in the hypolimnion demonstrated the lowest SR. The SI rarefaction curve reached a higher value for dimictic than polymictic lakes and is highest for the lowest trophic status lakes in all thermal zones. This pattern was most pronounced in the epilimnion (Fig. 7).

**Figure 8:**
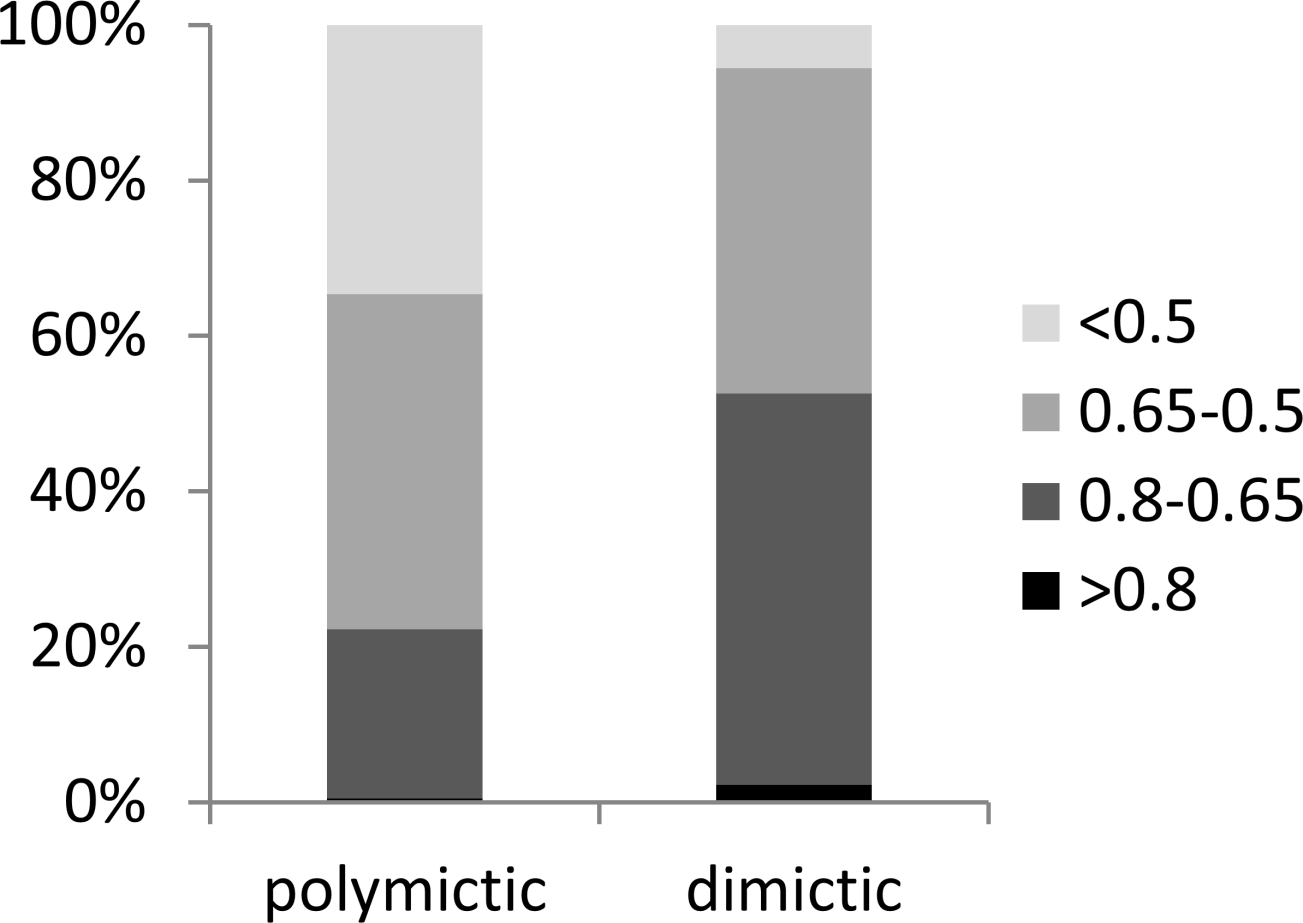
Zooplankton similarities for polymictic and dimictic lakes. Four levels of taxonomic similarity: <0.5—low degree of similarity; 0.5–0.65—moderate degree of similarity; 0.65–0.8—high degree of similarity; >0.8—very high degree of similarity.

In the dimictic lakes studied, a group of taxa (mainly Crustacea) that typically occur in deep lakes was found, some of which are not common in East-Central Europe (e.g., *Bythotrephes longimanus, Daphnia hyaline, Daphnia longiremis, Eubosmina longicornis*, *Heterocope appendiculata*)*,* or even globally (the rare glacial relict *Eurytemora lacustris*) (Table 3). In contrast, a few taxa considered typical for the littoral zone were found only in the polymictic lakes (e.g., *Brachionus quadridentatus, Lepadella quadricarinata, Simocephalus serrulatus*). We determined that polymictic lakes are more heterogeneous than dimictic lakes (which were homogenous) in terms of zooplankton composition. A high degree of similarity (0.8–0.65) was observed in 21% of polymictic lake pairs and 50% of dimictic lake pairs (Fig. 8). A low degree of similarity (<0.5) was observed in 34% of polymictic lake pairs and only in 5% of dimictic lake pairs.

**Figure 9:**
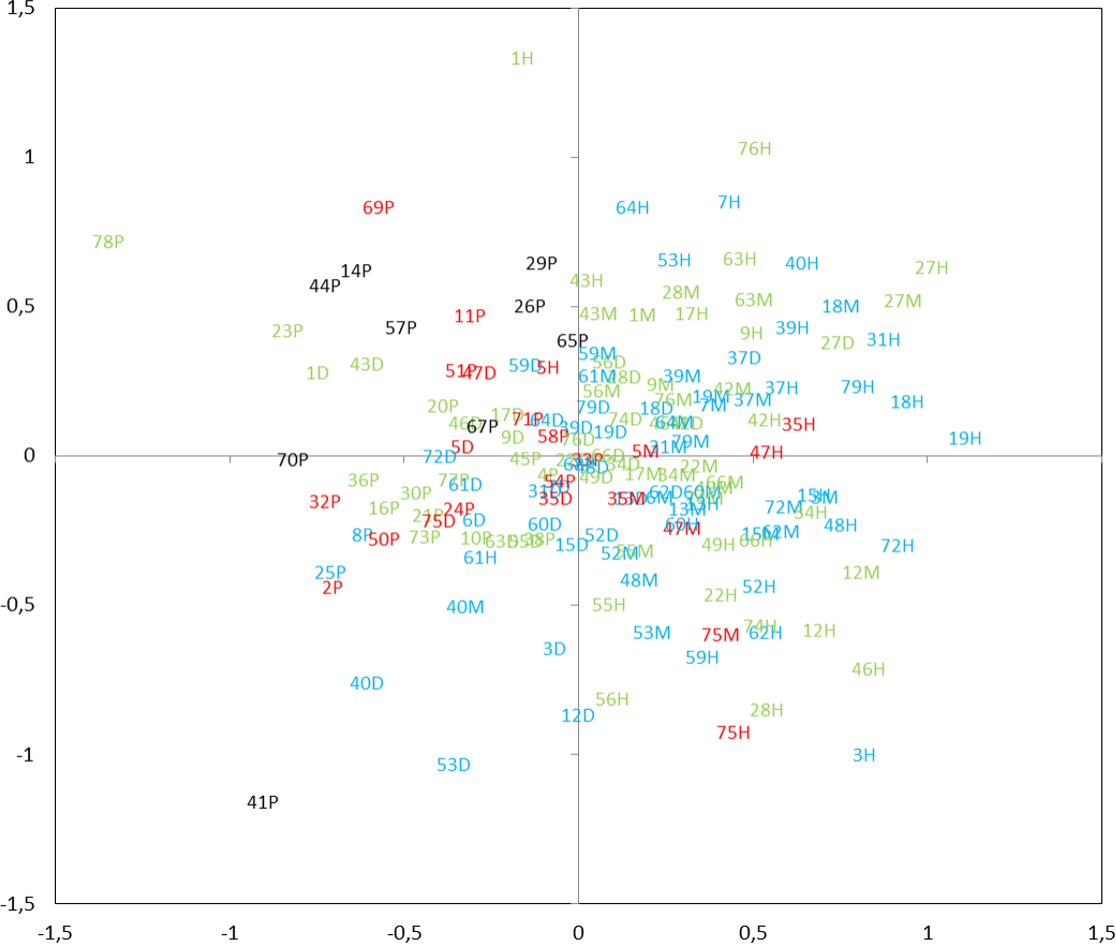
Non-metric multidimensional scaling (NMDS) plot displaying similarities of zooplankton within mictic type, thermal layers and trophic state index (TSI_SD_). Clusters of the different mictic types, layers (nP, polymictic epilimnion; nD, dimictic epilimnion; nM, dimictic metalimnion; nH, dimictic hypolimnion; *n*, the lake number, refer to Table 1) and the values of TSI_SD_ (TSI < 45—blue, TSI = 45–55—green, TSI = 55–65—red, TSI > 65 black )(stress = 0.27).

Ordination by NMDS showed clustering of the different mictic types, thermal layers, and the change in composition throughout the transparency profile (Fig. 9). The zooplankton communities in the metalimnion and the hypolimnion (of dimictic lakes) appeared to be very distinct from those in the epilimnion of shallow lakes. However, in many cases, the community of zooplankton in the epilimnion of dimictic lakes was more similar to those found of shallow lakes, than those found in deeper layers of dimictic lakes. The TSI_SD_ also had an influence on the zooplankton communities, which is demonstrated by the least-transparent lakes being clustered. Moreover, communities of several deep eutrophic lakes were similar to communities found in lower trophic level lakes.

## Discussion

### Impact of lake depth and mixing type on zooplankton biodiversity

In our study, the high number of rotifer species was crucial in shaping total zooplankton biodiversity because most species were rotifers. Many studies show that shallow waters are rich in microinvertebrates, especially in rotifer species (Karabin, 1985; Gilbert & Hampton, 2001; Gumiri & Iwakuma, 2002; Kuczyńska-Kippen, 2007; Ejsmont-Karabin, 2012; Pociecha et al., 2015), largely due because in shallow polymictic lakes, species (especially Rotifera) associated with the bottom or macrophytes can randomly be washed into the open water (Wallace et al., 2006; Ejsmont-Karabin, 2012). Wallace et al. (2006) stated that rotifer SR in water bodies with extensive vegetation could be more than three times higher than that in water bodies with small isolated vegetative patches (Grzybowski, 2014). Several authors have observed that, unlike in dimictic lakes, the habitat that macrophytes provide in shallow polymictic water bodies results in a high number of zooplankton species (Scheffer, 1998; Jeppesen et al., 1998; Kuczyńska-Kippen, 2007; Scheffer & Van Nes, 2007). Macrophytes colonize up to 100% of the area in polymictic lakes, whereas in dimictic lakes, plant distribution is limited to the littoral zone

Moreover, it appears that many rotifer species exhibit a preference for vertical placement in the epilimnion, as their SR was significantly lower in deeper layers of the dimictic lakes. Their greater abundance in the epilimnion can be explained by suitable physicochemical and trophic conditions of the water and behavioral cascade interactions (Berger, Diehl & Kunz, 2006; Gauthier, Prairie & Beisner, 2014). In turn, inverse relationships between rotifer and copepod biodiversity and lake depth could be caused by avoidance behavior of some rotifer species towards crustaceans. Gilbert & Hampton (2001) found the reverse daily migration of rotifer *Polyarthra remata* to be associated with the avoidance response of a small copepod *Tropocyclops* which migrated to deeper layers during the day to avoid being preyed upon by the predator. This relationship may also apply to cladocerans, which may also cause a decrease in rotifer diversity (Diéguez & Gilbert, 2011).

The higher number of species found in the deep lakes could be explained by the fact that the lakes encompass a greater range of environmental conditions, and so can accommodate more taxa (Lomolino, 2001). We found a similar pattern in terms of a greater number of copepod species in deeper lakes. The results of studies conducted with 47 shallow and deep water bodies in Italy (0.2–24 m) also indicate a positive relation between zooplankton communities and maximum water depth (Tavernini, Primicerio & Rossetti, 2009). Similar findings were reported by Keller & Conlon (1994) who determined that the species richness of crustaceans (Cladocera and Copepoda) was higher in deep Canadian lakes (maximum depth > 8 m) than in the shallow ones (maximum depth < 8 m). However, we have not found any relationship between lake depth and Cladocera species richness (despite similar trends in Copepoda). We assumed that in a heterogenous group of lakes in terms of depth, the Cladocera species richness would be homogenous due to different cladocerans replacing each other across a lake depth gradient. The general trend in this study was that large-bodied crustaceans occurred more in dimictic lakes than in polymictic lakes (e.g., *Daphnia* sp., *Cyclops abyssorum*, *Eudiaptomus* sp. *Eurytemora lacustris, Heterocope appendiculata, Leptodora kindtii, Megacyclops viridis).* This suggests that the mechanisms determining zooplankton biodiversity are different between shallow and deep lakes.

### Influence of TSI_SD_ on zooplankton biodiversity

When examining lake zooplankton biodiversity, researchers often find a unimodal peak in species richness at intermediate primary productivity (Dodson, Arnott & Cottingham, 2000; Waide et al., 1999; Barnett & Beisner, 2007). Hoffmann & Dodson (2005) argue that small catchment conversions (agriculture, urban, industrial, or residential development) have a positive impact on the SR of crustaceans because they increase the export of biogenic compounds from the catchment to the lake. Nevertheless, most lakes in Europe are affected by human activities (Jeppesen et al., 2000; Hoffmann & Dodson, 2005) and oligotrophic lakes are rare. Therefore, zooplankton species richness declines along the trophic gradient in Western European lakes (Jeppesen et al., 2000). Our study results show that only copepod biodiversity declines as the TSI_SD_ increases. On the other hand, we found a positive correlation between the rotifers and the trophic state index. In our study, most of the lakes with the highest index were polymictic (56% of polymictic lakes were classified as at least eutrophic whereas only 9% of dimictic lakes were classified as at least eutrophic), suggesting that shallower and consequently higher trophic status waters are associated with higher SR of rotifers and lower SR of copepods. Those relationships we found for mean values compared between lakes but regionally, trophic state could have a different impact on zooplankton biodiversity. To evaluate the influence of TSI_SD_ on zooplankton we applied rarefaction methods separately to all thermal zones.

It was found that the highest zooplankton SR and SI values were present in low trophic state lakes. However, there has been one exception of Crustacea, for which the highest Cladocera SR rarefaction curve value was detected in the epilimnion of eutrophic lakes. Concurrently, we determined that the SR value was greater in the epilimnion of dimictic lakes than that of polymictic lakes. There are some possible explanations for the low Cladocera SR in the epilimnion of transparent lakes. In low trophic status lakes, crustaceans migrate through deeper zones (Gliwicz, 1986) and cause impoverishment of the epilimnion, thus reducing the SR in the surface layer of a lake. Moreover, deeper lakes are resistant to eutrophication (Bajkiewicz-Grabowska & Mikulski, 2010), and in their deeper zones, there are cool waters which can hold more dissolved oxygen (Kajak, 2001). Furthermore, some environmental factors, such as the total amount of ammonia nitrogen, may not be toxic enough cause a zooplankton community shift in those lakes (Yang et al., 2017). It seems that in the deeper layers of clear-water lakes, environmental variables fluctuate very little and would be less important in shaping zooplankton biodiversity. Most species respond to significant amount of fluctuation (i.e., they constantly readapt to variable environments) (Fox, Nelson & McCauley, 2010), implying a larger number of species adapted to relatively stable conditions could have coexisted in the deep lakes during the period covered in this study. Relatively stable abiotic and biotic variables in lakes are necessary to maintain some populations which are found only in deep lakes, such as *Bythotrephes longimanus, Daphnia hyalina, Daphnia longiremis, Eurytemora lacustris, Heterocope appendiculata* (Kasprzak et al., 2005; Maier et al., 2011; Faustova et al., 2011; Błędzki & Rybak, 2016).

Many authors present only raw SR or SI data. Hence, their findings could have been different if the estimates were created using rarefaction methods. The SR and SI of zooplankton in eutrophic lakes could be high when examining individual lakes and comparing them separately. However, that is not the case when examining a substantial number of lakes grouped by trophic state. It appears that in eutrophic lakes, the high average number of zooplankton species in a single lake is often seen for the same species in each lake. Therefore, the rarefaction curve suggests that the group of lakes with the lowest trophic state is characterized by the highest SR. The different trophic states of lakes could also explain the higher value of SI in the dimictic lakes (rarefaction). Deep lakes are more resistant to eutrophication and pollution than shallow lakes (Hillbricht-Ilkowska, 2002), so it appears that the lower SI of zooplankton in shallow lakes was affected by natural stressors such as excess nutrients, limited dissolved oxygen, and rapid pH changes than in deep lakes, which led to the dominance of opportunistic species. This kind of fluctuating environment usually hosts a narrow spectrum of species and is often dominated by specialized species, while common taxa are supplanted due to unfavorable conditions (Horváth et al., 2014).

Zooplankton are usually sampled by vertical tows (i.e., raising a net through the water column) (Dodson, Arnott & Cottingham, 2000). The lake is divided into individual thermal zones, which, as indicated above, yielded different biodiversity values in various thermal zones. Consequently, it appears that the level of the zooplankton SR and SI (during daylight hours) depends greatly on the thermal zone, which also highlights the importance of the metalimnion in shaping zooplankton biodiversity. Numerous papers confirm that the metalimnion is crucial in shaping the crustacean zooplankton abundance and biomass (e.g., Gliwicz, 1986; Horppila et al., 2000; Armengol et al., 2012; Gauthier, Prairie & Beisner, 2014). However, we also stated that the metalimnion in temperate lakes provides an important niche for lake microcrustaceans, especially for Copepoda. On one hand, it provides stable temperature conditions, shelter (deep, dark waters), and, in the case of slightly eutrophic lakes, excellent oxygen conditions. The relationships between the depth, the TSI and the SR and the SI were often quite different. In addition, the inverse relation indicated that the optimum conditions for the pelagic biodiversity of rotifers and microcrustaceans were being shaped under different conditions. Consequently, greater rotifers richness can be observed in shallow waters, whereas microcrustaceans are more common in deep transparent waters. The multiple regression analysis explained a rather small percentage of the diversity in zooplankton SR and SI in the studied lakes, despite revealing significant correlations. We assume that other variables not taken into account (e.g., fish predation, food availability, thermocline depth, oxygen content, biotoxins, and the amount of ammonia nitrogen) could have affected the biodiversity of zooplankton (Gliwicz, 1986; Paerl et al., 2001; Armengol et al., 2012; Napiórkowska-Krzebietke & Hutorowicz, 2013; Gauthier, Prairie & Beisner, 2014; Yang et al., 2017)

### Similarity of zooplankton communities

Regarding the results of this work, and in the context of zooplankton taxonomic similarity between different lakes, it is worth examining the question posed by Jenkins & Buikema (1998) “Do similar zooplankton structures develop in similar environments?” Cottenie et al. (2003) have shown that combined and adjoining water bodies, with similar morphological parameters, are a potential habitat for meta-structures of local zooplankton communities. However, little is known whether the basic morphological characteristics that determine the mictic lake type will affect the taxonomic similarity between these lakes. The results of this study regarding the comparison of taxonomic similarity (polymictic–polymictic vs. dimictic–dimictic) show that low taxonomic similarity is more frequent among polymictic lakes than among dimictic lakes. As many as 52% of dimictic lake pairs were characterized by at least a high degree of taxonomic similarity, while only approximately 22% of polymictic lakes had such a degree of taxonomic similarity. This suggests that the composition of the pelagic species in the summer (Rotifera, Cladocera, and Copepoda) is more predictable in dimictic temperate lakes than in polymictic lakes, which in turn has an interesting implication for beta and regional diversity. Overlaying this pattern are those species that appear and disappear with different frequency (Scheffer et al., 2003; Wallace et al., 2006; Fox, Nelson & McCauley, 2010) that could be affected by proximity to egg banks in the sediment (Brendonck & De Meester, 2003), dispersal (Pinel-Alloul, 1995; Havel & Shurin, 2004) or local conditions. The most important determinants of high taxonomic similarity of zooplankton in deep lakes are the greater stability of water conditions and lower diversity habitats than in polymictic lakes (Scheffer & Van Nes, 2007). The importance of these factors can be confirmed by studies that examined the variability of the parameters of different lake types which suggest that polymictic lakes frequently change according to several parameters (e.g., Fee et al., 1994; Nõges, 2009). We observed a high degree of taxonomic similarity among the dimictic lakes in our study. In turn, this suggests homogeneity of the environmental conditions in these lakes. On the other hand, the low degree of species similarity among the polymictic lakes indicates a high degree of heterogeneity of shallow waters. Similar results were also found by Ejsmont-Karabin & Kuczyńska-Kippen (2001) who studied small urban water basins and reported that the similarity index showed a strong distinction between the Rotifer community structures. In the present study, both lake types presented a group of species that appeared very frequently (e.g., *Keratella cochlearis*, *Polyarthra vulgaris*, *Mesocyclops leuckarti*, *Trichocerca similis*, *Daphnia cucullata*, *Diaphanosoma brachyurum*, *K. cochlearis v. tecta*, and *Thermocyclops oithonoides*), nevertheless the differences between zooplankton assemblages in polymictic vs. dimictic lakes were pronounced. By using the non-metric multidimensional scaling (NMDS) method, it was found that the zooplankton communities among the mictic types were significantly different, which was closely associated with thermal layer and trophic state. The most crucial factor in shaping zooplankton communities was the mictic type. Consequently, deeper layers of dimictic lakes were very distinct from the epilimnion of shallow lakes. However, in many cases, communities in the epilimnion of deep lakes were more similar to those of shallow lakes than those of deeper layers of dimictic lakes. This was because the deeper layers allow the development of distinct communities of zooplankton, as previously indicated by the lower Rotifera diversity of and greater Crustacea diversity. Therefore, taxonomic composition was related to environmental conditions, which is supported by the mechanistic explanation for the trends observed. Those significant differences between mictic types should be taken into consideration in ecological research as well as in the conservation of freshwater organisms. This means that both mictic lake types are important places of zooplankton biodiversity which vary in quality as the biodiversity levels shift among rotifers and microcrustaceans. Moreover, the zooplankton biodiversity is shaped in response to depth and trophic state.

## Conclusion

Polymictic lakes are characterized by having a higher average species richness than dimictic lakes, which is attributed to a large number of Rotifera species in shallow waters. Shallow lakes are usually more eutrophic, yet as they lose their thermal niches (metalimnion and hypolimnion), they gain macrophyte niches or gain proximity to the substrate, which together causes a decrease in Copepoda biodiversity and increase in Rotifera biodiversity. However, at a regional scale (using rarefaction), the highest species richness and Shannon biodiversity values are found in Cladocera, Copepoda and Rotifera in non-eutrophic waters. This finding indicates a large number of zooplankton species in the low trophic waters of the studied region. Moreover, polymictic lakes are characterized by higher variability of zooplankton composition than dimictic lakes.

##  Supplemental Information

10.7717/peerj.5731/supp-1 Supplemental Information 1 Data used for epilimnion analysis

10.7717/peerj.5731/supp-2 Supplemental Information 2 Data used for metalimnion analysis

10.7717/peerj.5731/supp-3 Supplemental Information 3 Data used for hypolimnion analysis

## Additional Information and Declarations

### Data Availability

The following information was supplied regarding data availability: The raw data are provided in the Supplemental Files.
